# Recent Advances in Antibody Discovery Using Ultrahigh-Throughput Droplet Microfluidics: Challenges and Future Perspectives

**DOI:** 10.3390/bios15070409

**Published:** 2025-06-25

**Authors:** Dhiman Das, John Scott McGrath, John Hudson Moore, Jason Gardner, Daniël Blom

**Affiliations:** Ampersand Biomedicines Inc., Southline Suite 3A, 135 William T. Morrissey Blvd., Boston, MA 02125, USAjmcgrath@ampersand.bio (J.S.M.);

**Keywords:** antibody discovery, droplet microfluidics, FADS, binding assay, FRET assay, internalization assay, neutralization assay, “hit” droplet, sorting workstation

## Abstract

Droplet microfluidics has emerged as a transformative technology that can substantially increase the throughput of antibody “hit” discovery. This review provides a comprehensive overview of the recent advances in this dynamic field, focusing primarily on the technological and methodological innovations that have enhanced the antibody discovery process. This investigation starts with the fundamental principles of droplet microfluidics, emphasizing its unique capabilities for precisely controlling and manipulating picoliter-volume droplets. This discussion extends to various assay types employed in droplet microfluidics, including binding assays, functional assays, Förster Resonance Energy Transfer (FRET) assays, internalization assays, and neutralization assays, each offering distinct advantages for antibody screening and characterization. A critical examination of methods to improve droplet encapsulation is presented, besides addressing challenges such as reducing the leakage of small molecules from droplets and explaining what a “hit” droplet looks like. Furthermore, we assess design considerations essential for implementing high-throughput fluorescence-activated droplet sorting (FADS) workstations and emphasize the need for automation. This review also delves into the evolving commercial landscape, identifying key market players and emerging industry trends. This review paper aims to catalyze further research and innovation, ultimately advancing the field towards more efficient and robust solutions for antibody identification and development.

## 1. Introduction

Monoclonal antibodies (mAbs) have become one of the most successful and versatile classes of biological drugs, with over 200 therapeutic antibodies currently on the market and nearly 1400 investigational candidates undergoing clinical evaluation [1,2]. Antibody engineering for optimized and novel functionalities provides unprecedented opportunities in our quest to develop innovative treatments for various diseases. Antibodies are used to specifically bind and neutralize their targets, such as cancer cells [3] or disease-causing pathogens [4]. This allows for targeted therapy, where the antibodies can block the activity of these target molecules, such as IL-5, cMET, RSV, or SARS-CoV2, neutralize toxins, or recruit the immune system to attack specific cells [1]. This has revolutionized the treatment of various diseases, including cancers [3,5,6], autoimmune disorders [7], and infectious diseases [8]. As of November 2024, custom-designed antibodies, including antibody-drug conjugates (ADCs), bispecific/multispecific antibodies, and other engineered formats, represent approximately 25% of the approved antibody products and around 30% of investigational antibody therapeutics under regulatory review [2].

Antibody discovery utilizes a combination of in vivo [9], in vitro [10], and in silico [11] approaches to generate antigen-specific variants. The in vivo approach, one of the earliest methods, firstly involved fusing mouse myeloma cells with immunized mouse spleen cells to create hybridoma cell lines that produce monoclonal antibodies. Recent advances in this approach include using transgenic mice to enable humanized mAb production [12,13], generating antibody sequences from peripheral blood mononuclear cells (PBMCs) isolated from whole blood samples [14] and human B cell receptor (BCR) repertoires mining [15]. Recently, in vitro methods like phage and yeast display have dominated antibody screening efforts. These platforms involve expressing whole antibodies or antibody fragments (e.g., scFv or Fab) on surfaces of bacteriophages or yeast cells and selecting them based on binding specificity and functional impact [10] using techniques such as fluorescence-activated cell sorting (FACS) [16,17]. While effective, these methods often fail to reflect the natural in vivo pairing of antibody heavy and light chains, which may result in antibodies with undesirable properties. To address this limitation, direct screening for in vivo mAbs from B cells participating in natural immune responses is increasingly preferred.

High-throughput screening (HTS) technologies are critical for efficiently identifying antibodies of interest. Beyond their throughput and cost-effectiveness, their key advantage lies in targeting difficult antigens with low antigenicity, facilitating the screening of rare mAbs. This capability is enhanced by synergizing with generative artificial intelligence (AI) [18,19]. This synergy addresses previously intractable targets and accelerates therapeutic development by integrating generative AI’s design capabilities with HTS’s validation power. Generative AI rapidly produces a vast array of potential compounds or antibodies, focusing on characteristics suited to challenging or rare targets, thereby expediting the initial stages of discovery with a highly targeted library. HTS swiftly evaluates these AI-designed theoretical candidates against biological targets, identifying promising leads with high precision and avoiding the lengthy trial-and-error process typical of traditional methods [9,20,21,22]. Moreover, feedback from HTS informs AI algorithms, allowing continuous refinement of candidate molecules via traditional methods. This iterative process efficiently refines potential leads, significantly reducing the discovery and validation timeline.

Microarray systems [23] have laid the groundwork for large-scale screening efforts but often face bottlenecks in throughput and cost-effectiveness. A significant limitation of these microstructure arrays is that they have a fixed number of confinement locations, which restricts the number of cells that can be screened per experiment. Some platforms can screen tens of thousands of cells per run, yet given that the proportion of B cells with specificity to antigens can be as low as 0.05%, millions of B cells must be screened to find those with the desired properties, presenting significant logistical and financial challenges for research programs [24].

Other innovative microarray-based platforms can accommodate millions of cells in individual culture wells of volume 1 nL each [25]. However, as the microwells are considerably larger than the cells, the single-cell seeding efficiency is governed by Poisson statistics [23]. Also, the single-cell trapping mechanism in these microstructures is driven by gravity. After cells have been seeded in the microstructures via sedimentation, the excess cells are washed away by pipetting, which often causes the seeded cells to be easily dislodged [26]. There is considerable potential to refine these methods, enhance single-cell seeding efficiency, and optimize the overall effectiveness of microstructure arrays for large-scale cell screening. Collaborative advancements could lead to more cost-effective and high-throughput solutions in this field.

Droplet microfluidics has emerged as a transformative technology for high-throughput screening (HTS) by enabling the ultra-high-throughput encapsulation of individual cells or particles, such as B cells, yeast cells, or phage particles, into discrete droplets at kiloHertz rates [27,28,29]. This technique allows for the execution of millions of assays within hours, preserving sample integrity and minimizing cross-talk between reactions. Unlike display technologies (phage/yeast), which disrupt native heavy-light chain pairing, droplet microfluidics facilitates single-cell resolution analysis, preserving native antibody pairing, capturing rare clones, and effectively capturing B-cell diversity. It can also be integrated with reverse transcription-polymerase chain reaction (RT-PCR) or next-generation sequencing (NGS) technologies [30]. This integration allows for the efficient sequencing of genetic material from individual cells or clones based on their phenotypes, providing detailed insights into the genetic basis of functional properties. Furthermore, it enhances the specificity and sensitivity of antibody screening by enabling the precise control of microenvironments surrounding individual B cells through the use of antigen-coated beads, which increases the signal-to-noise ratio (SNR) [28,31]. A significant benefit is its high co-encapsulation efficiency, which ensures that cells and assay reagents are encapsulated with precision, improving experimental reproducibility while reducing sample consumption and associated costs [29]. One of the key advantages of this technology is its ability to detect low-abundance hits, as the encapsulation of individual cells in picoliter-sized droplets reduces dilution and enhances signal detection, thereby increasing the sensitivity for identifying rare antibodies [32]. These advantages have made droplet microfluidics an invaluable tool in both academic research and industrial applications, where it has been used to increase rare antibody identification, reduce cycle time, and streamline workflows for antibody discovery and monoclonality checks.

This review paper explores recent advances in antibody discovery using droplet microfluidics. It discusses challenges associated with current technologies, highlights the advantages offered by droplet-based systems, and provides future perspectives on their integration into high-throughput workflows for therapeutic antibody development.

## 2. Fundamentals of Droplet Microfluidics

**Figure 1:**
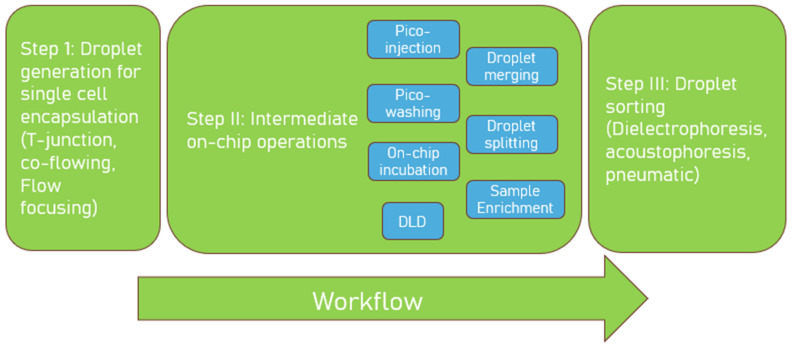
Overview of the various on-chip microfluidic operations or capabilities that can be used at single-cell resolution for antibody discovery. DLD = Deterministic lateral displacement.

The initial step in droplet microfluidics entails encapsulating reagents, including cells and assay components, within monodisperse droplets utilizing droplet generation chips. In the interim, various on-chip, droplet-based microfluidic configurations can be included and incorporated to perform intermediate operations tailored to specific assay requirements. These microfluidic operations can include sequentially adding or removing reagents from droplets, on-chip incubation, and more, as detailed in Section 2.3. Figure 1 summarizes the diverse on-chip microfluidic operations and capabilities for high-throughput antibody discovery at single-cell resolution. The final step involves screening and sorting these droplets according to signal intensity, a function of the biological response between the assay components. This step is facilitated by sorting chips.

### 2.1. Droplet Generation Chips and Parallelization

Generally, most microfluidic devices are made of polydimethylsiloxane (PDMS) in the prototyping stage [33]. Other than PDMS, commonly used materials include glass, polymethyl methacrylate (PMMA), cyclic olefin copolymer (COC), etc. [34]. For droplet generation, devices with a T-junction, flow-focusing junction, or co-flowing junctions [27] generate monodisperse aqueous droplets in oil. These aqueous droplets are regarded as the dispersed phase, and the external oil phase is also known as the continuous phase. The droplet sizes and the generation frequencies are dictated by the flow rate ratios of the continuous and dispersed phases and the channel geometry. The generation frequency of these droplets is in the range of a few kiloHertz [35], which enables the rapid encapsulation of cells and other entities into droplets. Typically, the encapsulation efficiencies of single cells and beads into droplets are dictated by Poisson statistics [36]. Recently, curved microchannels have been employed to generate secondary vortices (Dean vortices), which can manipulate the position and mixing of particles or fluids within the channel to enhance the co-encapsulation of multiple particles such as cells and beads, within droplets [37]. A more detailed explanation of this strategy has been provided in Section 4.1 under “New Developments for High Throughput Screening”. Other strategies include increasing the droplet generation rates or frequencies by coupling several T or flow-focusing junctions in parallel [38,39], either in a single layer or by using a step emulsification method [40]. In general, high-throughput droplet generation rates have been achieved using several integrated droplet formation junctions sharing the same inlets and outlets. The microchannel geometries were designed to split the fluid streams symmetrically, ensuring equal distribution of the dispersed and continuous phases to each junction.

### 2.2. Intermediate On-Chip Microfluidic Operations to Achieve On-Chip Workflow Functionality

#### 2.2.1. Pico-Injection and Pico-Washing

Pico-injection was first reported in 2010 by David Weitz’s group [41]. It is a robust technique for adding controlled volumes of reagents to the microfluidic droplets using an electric field at a throughput of kilohertz rates. An electric field was applied to the pico-injection nozzle, destabilizing the water/oil interfaces and allowing the reagent to enter the droplet. This operation can also perform multiple injections for serial and combinatorial additions. Previously, pico-injection enabled the detection of RNA transcripts by adding TaqMan probes and sequential delivery of viruses to host cells in droplets [42,43]. This method could also select and encapsulate cells and beads into droplets on-demand [44]. Recently, it was shown that it is possible to control the injection volumes into each droplet [45]. First, a laser beam excites each droplet, and the fluorescence intensity is measured. Then, the time delay between droplet detection and the electric field is adjusted to control the injection volume. Recently, Alexis et al. [31] conducted fluorescence-activated droplet sequencing (FAD-seq). In this process, droplets containing cells with a specific fluorescent phenotype are selectively pico-injected with RT-PCR reagents and sorted using dielectrophoresis (DEP). The light and heavy chain genes are then naturally paired, fused into a single-chain fragment variant format, and amplified before being transferred off-chip for subsequent nanopore sequencing. A similar droplet microfluidic operation is pico-washing, which can exchange the fluids within the droplets with a different fluid at kiloHertz rates [46]. This technique allowed simultaneous fluid addition and removal for continuous flow washing of the contents from within microfluidic droplets.

#### 2.2.2. On-Chip Incubation

For long reaction times (>1–2 h), the droplets can be incubated within an on- or off-chip reservoir and reinjected into the microfluidic device for analysis. However, when the on-chip incubation times are within 1 min to an hour, delay lines [47] can be used, which allow for precise incubation times and efficient droplet handling. The usage of delay lines required several additional features [47], such as fabricating (i) a two-layered microfluidic device with taller, wider channels and (ii) oil extraction structures to reduce back pressure and facilitate longer incubation times. Oil extraction structures were helpful in increasing the packing density of the droplets. Finally, (iii) constrictions were also necessary along the delay line to reduce the variation in incubation times. Ideally, all droplets should have the same incubation time to ensure consistent and accurate reaction results within the droplets. However, due to factors like parabolic flow profiles within the channels, droplets travel at different speeds depending on their position. This leads to some droplets having longer or shorter incubation times than others. Reducing this variation in the incubation time helps to provide more reliable and precise measurements in droplet-based microfluidic assays; hence, constrictions were designed along the channel width of the dispersion line, causing the droplets to be stochastically redistributed at each constriction. However, the order of the droplets was not maintained in that work. Recently, researchers have also used hydrodynamic modeling [48] to optimize droplet clustering and incubation efficiency. These advancements enable high-throughput and reliable droplet incubation on microfluidic devices.

#### 2.2.3. Deterministic Lateral Displacement (DLD)

Microfluidic droplets loaded with single B cells and specific stimuli such as antigens, cytokines, etc., require incubation for antibody secretions. Depending on the rates of cell secretions, this may result in droplets losing their monodispersity due to cell metabolism altering the composition of the inner droplet. This can cause osmotic pressure differences across the droplet interface [49]. Moreover, the high flow rates in the sorting chips can also induce shear stress, which can destabilize the droplets [50]. If the droplets lose their monodispersity, the re-injected droplets are no longer equidistant in the sorting chips, negatively impacting the sort pulse duration and reducing sorting efficiency. DLD is a passive particle separation method in a microfluidic device known for its ability to separate particles based on their size with a tolerance of <10 nm [51]. This technique employs a regularly spaced array of micropillars to generate a specific streamlined pattern. Particles with a size exceeding a certain critical diameter, Dc, are sidetracked in each row and travel along a predictable trajectory through the pillar array (known as displacement mode). Conversely, particles smaller than Dc adhere to the laminar streamline paths within the pillar array (zigzag mode). Due to this, droplets of different sizes can be separated into different collection outlets, and monodispersity can be enhanced. Tottori et al. [52] demonstrated the separation of main droplets from satellite droplets using DLD. Apart from DLD, pinched flow fractionation (PFF) is another method for the size-dependent separation of droplets [53,54].

#### 2.2.4. Droplet Merging: Passive and Active (Electrocoalescence)

The discrete nature of microfluidic droplets within the oil phase restricts direct physical access to their contents on-chip, posing challenges for integrating microdroplets with other platforms, such as mass spectrometry, capillary electrophoresis, and liquid chromatography. Electrocoalescence technology [55] offers a solution by enabling the extraction of microdroplet contents and incorporating them into a continuous aqueous stream. This is achieved by inducing droplet coalescence with an aqueous stream via the application of an electric field across the channel. The process is controlled by applying voltage across the microchannel, allowing for continuous or discrete operation. The discrete collection of droplets is managed by an external electrical signal, which can be triggered on demand based on the droplet contents. Ali et al. [53] demonstrated the use of an electrocoalescence-based microfluidic device to enrich lacZ genes, achieving a 502-fold enrichment from a 1:100 molar ratio of lacZ to lacZmut genes, with a throughput of 2000 droplets per second.

#### 2.2.5. Droplet Splitting

Droplet splitting offers the opportunity to perform multiple assays on a single droplet. This process divides a droplet into smaller “daughter” droplets, allowing its contents to be shared throughout all of those daughter droplets. For instance, on the ESI-Mine platform, droplets were split into two so that one daughter droplet was analyzed by mass spectrometry, and the other droplet entered a sorting module for inline retrieval of “hits” [56,57]. In the context of antibody discovery, ESI-Mine can analyze mass spectrometry data to confirm the presence of specific antibodies by identifying and characterizing the proteins and peptides that make up the antibody structure. Moreover, ESI-Mine’s integration with advanced software tools may enhance its capabilities in providing insights into detailed amino acid sequencing, extending beyond the initial identification typically achieved with mass spectrometry. Typically, the droplets are coated with surfactants, making it difficult to split them. The earliest study demonstrated droplet splitting without utilizing surfactants, achieving the splitting through the collision of droplets at a microfluidic T-junction [58]. Recently, novel strategies have been incorporated for droplet splitting even in the presence of surfactants [59,60]. One approach employed multi-furcating microfluidic channels, where the splitting process was influenced by the size of the mother droplets and the flow resistance of the furcating channels [59]. Another strategy utilized a dielectrophoretic (DEP) force to move all cells to one side of a droplet, followed by asymmetrical splitting to obtain a tiny daughter droplet containing all or most of the cells [60].

#### 2.2.6. Sample Enrichment by Ion Enrichment

Sample enrichment can be essential in those workflows where it becomes crucial to selectively increase the concentration of target proteins, making them more detectable and improving assay sensitivity. Microfluidics-enabled concentration polarization (CP) is an active research area with great potential for sample enrichment in microfluidic devices [61,62,63]. This technique operates based on the principle of selective ion transport, which can be leveraged to concentrate analytes of interest in a sample. This is performed by creating an ion depletion zone that initially repels charged particles or species and eventually leads to their accumulation at the boundary. Zheng et al. [61] displayed the capability of CP by electrokinetically concentrating fluorescein isothiocyanate-bovine serum albumin (FITC-BSA) with an initial concentration of 0.002 ng/mL and a concentration factor of a hundred million-fold enrichment was later achieved. Park et al. [63] reported a combination of the CP-based preconcentration and dielectrophoresis (DEP) methods through which the binding of fluorescein-tagged avidin to biotin-coated polystyrene beads was achieved. The binding of avidin to the biotin-conjugated particles was quantified by measuring the resulting fluorescence intensity. The combination of CP-based preconcentration and DEP trapping significantly increased the detection sensitivity of bead-based immunoassays. CP preconcentrated biomolecules and functionalized nanoparticles, while DEP trapped the preconcentrated particles, leading to a higher concentration of target molecules at the detection site.

### 2.3. Sorting Chip

Sorting chips are designed to separate and isolate droplets based on various physical or biochemical properties. Droplets containing multiple cells or merged/satellite droplets are generally excluded. Only droplets containing a cell(s), which is a high producer of specific antibody molecules, should ideally be sorted by these sorting chips. These chips include at least two inlets, one used for injecting emulsions and the other (one or more) for infusing spacer oil, which spaces out the droplets equidistantly. The inter-droplet distance needs to be consistent for maintaining the different process parameters such as sort pulse amplitude, sort pulse duration, etc., which, in turn, activates the external forces needed to sort the droplets reliably into the collection outlet. Various kinds of external forces [64] such as dielectrophoresis (DEP) [65], acoustophoresis [66], or pneumatic forces [67], are used to deflect the droplet of interest toward the collection outlet. However, the most robust and commonly reported droplet sorters in microfluidics are based on an electric field, which has reported the highest sorting rates up to 30 kHz (droplet volume = 6 pL) [68]. For the rational design of a high-throughput sorter, Simon et al. [65] compared different DEP-based sorter designs published over the past decade, performed numerical simulations to characterize several electrode designs’ performance and developed different design guidelines based on optimizing DEP force-acting on the droplets at the sorting junction. On a separate note, there is a trade-off between the droplet volume and sorting throughput reported by Isozaki et al. [69] and his work achieved a record throughput of 4.4 kHz for large droplets (droplet volume = 100 pL) by sequentially activating and deactivating driving electrodes. This was performed by synchronized electrode activation corresponding to the speed and position of a passing target droplet. Traditionally, sorting chips can only perform binary sorting as they contain only one collection outlet and one waste outlet. But recently, sorting chips with multiple collection outlets have also been reported, which can be used to further distribute the positive “hit” droplets of interest into different bins for complex multi-parameter sorting [70,71,72]. To reduce the number of false positive hits, novel designs [73] have also been implemented to eliminate the polydisperse droplets formed by unintentional droplet coalescence and splitting from the sorting regions.

## 3. Types of Assays

### 3.1. Binding Assays

**Figure 2:**
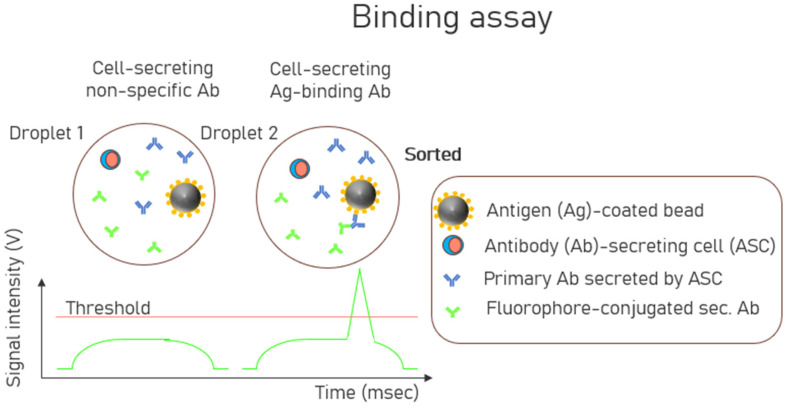
Schematic of a bead-based binding assay format where Droplet 1 contains Antibody (Ab)-secreting cells (ASCs) secreting non-specific antibodies and Droplet 2 contains ASCs which secret antigen(Ag)-specific antibodies. Only Droplet 2 will be sorted due to its signal intensity being above the threshold due to the localization of the fluorophore-conjugated secondary antibodies (Ab) on the beads.

The first fluorescent-activated droplet sorting (FADS) [74] method was reported in 2013 to analyze the detection of strong Ag-Ab affinity at a single-cell resolution within droplets. The binding of fluorescently labeled secondary detection Abs to the captured antibodies concentrates the fluorescence signal on the beads. The more antibodies the cell secreted, the more fluorescent probes bound to the beads, resulting in a higher fluorescence intensity. If the fluorescence intensity of the droplet exceeded a preselected threshold, the sorting electric field was turned on, resulting in a DEP force that deflected the droplet into a collection channel. A schematic of this droplet-based assay format is shown in Figure 2. Mazutis et al. [74] described droplet encapsulation with three entities: (1) Ag-coated capture beads, (2) Ab-secreting cells (ASCs), and (3) secondary fluorescently labeled detection antibodies. Throughout the incubation period, the secreted Abs from the ASCs were trapped by the capture beads, leading to the localization of the fluorescently labeled detection antibodies on the beads. This made the beads more fluorescent than those in neighboring droplets containing non-secreting cells within the microfluidic emulsion to generate detectable signals. Another work used a high-throughput microfluidic single-cell cytokine detection method for detecting cytokine (IL-2, IFN-γ, TNF-α) secretion of single, activated T-cells in agarose droplets over time [75]. Cytokines are signaling molecules that play a critical role in the immune system. They are primarily produced by specific immune cells, particularly T cells and natural killer (NK) cells, in response to stimulation by antigens, such as viruses, bacteria, or tumor cells. The collected droplets were cooled to 4 °C to facilitate gelation. Subsequently, cytokine secretion was examined in gel beads containing stimulated Jurkat T cells and cytokine-capture beads, and the results were compared to those from gel beads containing non-stimulated Jurkat T cells.

### 3.2. Functional Assays

**Figure 3:**
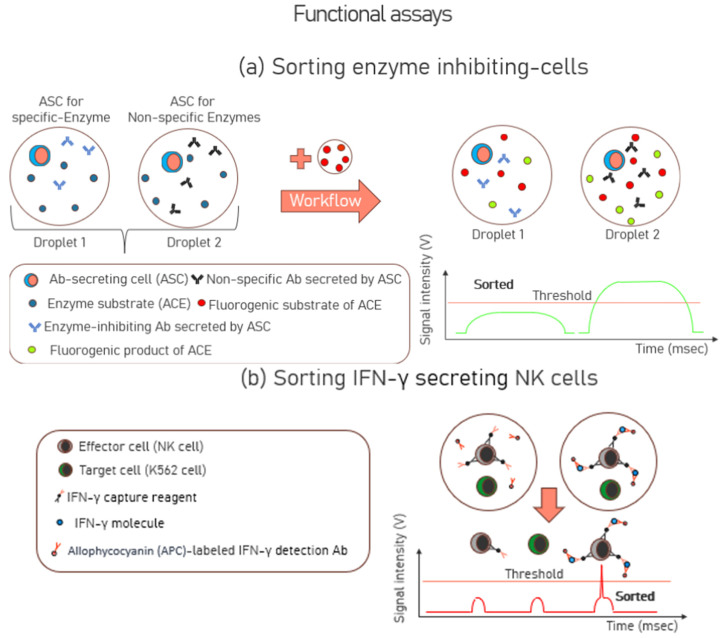
Schematic of functional assays where (**a**) sorting is performed to isolate enzyme-secreting antibodies. Only Droplet 1 would be sorted due to its lower signal intensity below the threshold due to the lower concentration of the fluorogenic product of angiotensin-converting enzyme (ACE). (**b**) Effector cells were encapsulated and incubated with target cells to activate them for secreting IFN-γ molecules. Only the effector cells coated with fluorescently labeled IFN-γ detection antibodies and those above the signal threshold were sorted. A commercial Fluorescence-Activated Cell Sorting (FACS) system performed the sorting.

Unlike binding assays that detect the presence or quantity of a molecule based on binding affinities, functional assays assess the biological activity or physiological effects of Abs on the target entities such as fluorogenic substrates or reporter cells. In-droplet functional screening was demonstrated for the first time in 2012, using hybridoma cells, which secreted the monoclonal antibody, 4E3, to inhibit the activity of angiotensin-converting enzyme 1 (ACE-1) [76]. The inhibition of ACE-1 is crucial as it serves as a key therapeutic target for the treatment of hypertension and congestive heart failure. Cells expressing 4E3 antibodies were spiked into an unrelated hybridoma cell population in a ratio of 1:10,000 and were encapsulated in droplets, together with the enzyme ACE-1. Next, these droplets were injected with a fluorogenic substrate of ACE-1. Only those unrelated hybridoma cells whose antibodies had no specificity to ACE-1 had an excess of ACE-1 available, which converted the fluorogenic substrate of ACE-1 to its fluorogenic product. Droplets showing low fluorescence intensities (indicating a low ACE-1 activity) were sorted, and a 9400-fold enrichment of the cells expressing 4E3 was observed. Sorted drops were broken by adding an equal volume of 1H, 1H, 2H, 2H-Perfluoro-1-octanol, and subsequently, hybridoma cells were isolated and seeded into 96-well plates. After expansion, the supernatants were characterized by ELISA for their antibody concentration. The workflow schematic of this functional assay is shown in Figure 3a.

To investigate the secretion of Interferon-gamma (IFN-γ), another study employed a functional approach by incubating effector cells, specifically natural killer (NK)-92 MI cells, with target cells derived from the K562 human leukemia cell line. This co-culture was conducted within droplets, allowing researchers to closely examine the interaction between these cells and the resultant IFN-γ secretion dynamics [77]. NK cells play a crucial role in the immune system due to their ability to secrete IFN-γ, a critical cytokine that activates macrophages. Natural killer (NK) cells, when exposed to K562 cells, become activated and acquire the ability to phagocytose and destroy various pathogens, including infected or cancerous cells. The NK-92 MI cell line is frequently used as a model system in research because it retains many of the key characteristics and functions of primary NK cells, making it ideal for in-depth study. In this context, NK cells were activated by encapsulating them with K562 cells, as these cells often present ligands that engage the activating receptors on NK cells. Before encapsulation, the NK cells were coated with IFN-γ capture reagents. Once the NK cells started secreting IFN-γ post-encapsulation and incubation, the IFN-γ were captured on the cell surface of the NK cells. The droplets were also encapsulated with fluorescently labeled IFN-γ detection antibodies, which allowed for the identification of the activated NK cells. Following in-droplet incubation, the cells were recovered from the droplets, and FACS efficiently sorted individual activated immune effector cells. Compared with corresponding assays performed in bulk, droplet compartmentalization greatly eliminated contamination between cells and reduced false negatives and positives. A schematic of this functional assay is shown in Figure 3b.

Wang et al. [78] detailed other novel functional screening strategies to identify bispecific T cell engager (BiTE) and agonist antibodies for next-generation cancer immunotherapy. Human Epidermal Growth Factor Receptor (HER)2 is a protein that functions as a receptor to and is often upregulated on the surface of specific cancer cells, particularly breast cancer. Infected K562-HER2 cells expressing the anti-HER2 × anti-CD3 BiTE positive control were encapsulated with reporter cells in droplets for screening bi-specific antibodies. The reporter cells used were Jurkat/pIL2-eGFP cells derived from the Jurkat cell line, a human T-cell leukemia used in the study of T-cell biology, signal transduction, and leukemia. When Jurkat cells were transfected with a vector carrying the enhanced green fluorescent protein (GFP) under the control of the full-length IL2 promoter, these cells expressed GFP when stimulated with CD3. Once stimulated (activated), the GFP-expressing reporter cells were fluorescently detected, which allowed for sorting. The study identified five bispecific antibodies among the sorted droplets, with BiTE1, BiTE2, and BiTE3 demonstrating successful activation of the reporter cell in the presence of K562-Her2. In vitro cytotoxicity assays showed that BiTE1 specifically induced the lysis of HER2-expressing SK-BR-3 breast cancer cells, showcasing antitumor activity. Further validation with flow cytometry revealed that BiTE1 stimulated expression of the activation marker CD69 on T cells, with dose-dependent increases in the secretion of interferon-γ (IFN-γ) and interleukin-2 (IL-2) observed in the supernatants.

**Figure 4:**
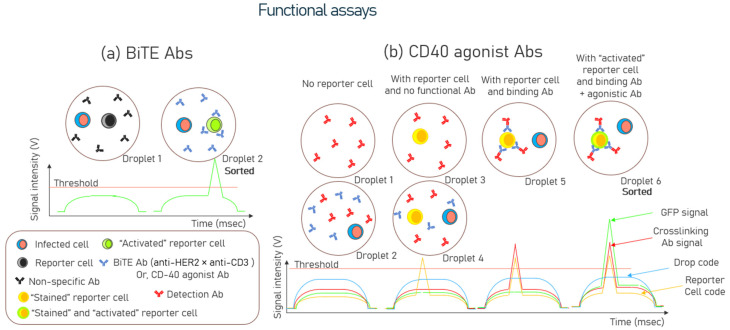
Schematic of functional assays where (**a**) sorting is performed to isolate bispecific T cell engager (BiTE) antibodies. Only Droplet 2 would be sorted due to the presence of the GFP signal of the “activated” reporter cell, (**b**) sorting is performed to isolate CD40 agonist antibodies. There are six possible signal intensity-based voltage traces. Only Droplet 6 would be sorted due to the overlap of the GFP signal, localization of cross-linking detection antibodies, reporter cell’s code, and the droplet code.

Similarly, CD40 agonist antibodies were sorted using infected HEK293 FT cells co-expressing red fluorescent proteins (RFP) and CD40 ligands(L) from a spiked library. Infected HEK293FT cells were sorted for CD40-agonist antibodies transfected with a phage display-based antibody library constructed using PBMCs from 30 healthy donors. These HEK293 FT cells were encapsulated with Jurkat T cells, which could express GFP when CD40 was activated. Droplets containing activated reporter cells with the localized GFP signal were sorted, and antibody genes from sorted cells were sequenced to identify and analyze the functional antibodies. The functional screening of CD40 agonist antibodies increased the percentage of the CD40L protein-secreting HEK293 FT cells to 51.94% from the input of 1.24% after droplet sorting. Finally, one of the scFv genes, C04, amplified from the sorted cells, displayed antitumor activity comparable to CP-870,893 (Pfizer), the most potent CD40 agonist antibody among those studies in clinical trials. Schematic illustrations of BiTE Abs and CD40 agonist Abs are shown in Figure 4a and Figure 4b, respectively.

### 3.3. Förster Resonance Energy Transfer (FRET) Assays

Droplets in a FRET assay provide a robust and efficient platform for high-throughput screening and sorting of ASCs. FRET describes energy transfer between two light-sensitive molecules [79]. When one molecule (donor) absorbs light, it can transfer some of its energy to another nearby molecule (acceptor) without emitting a photon. This transfer depends on the overlap between the donor’s emission spectrum and the absorption spectrum of the acceptor and their physical proximity. FRET has been used to analyze and sort ASCs at high throughputs against specific targets of interest [80,81,82]. Before implementing a FRET assay in droplets, a bulk mixture of donor and acceptor molecules with specific/non-specific antibodies was evaluated for efficiency and selectivity in microtiter plates [81]. Once the assay was established, a calibration curve was generated to determine the concentration of secreted antibodies over time. Subsequently, individual cells were encapsulated in droplets along with the FRET assay reagents, including the FRET donor and the FRET acceptor pair, to sort the cells that were high secretors of antibodies. The FRET assay mix in the droplets contained cell growth medium, Alexa Fluor 488-labeled secondary antibody (FRET donor), and Alexa Fluor 647-labeled c-myc peptide (FRET acceptor). Post encapsulation, the droplets are incubated to allow the cells to secrete antibodies. If a cell secreted the target antibody, which was anti-c-myc antibody, it brought the Alexa Fluor 488-labeled secondary antibody (FRET donor), and Alexa Fluor 647-labeled c-myc peptide (FRET acceptor) in close proximity, enabling the FRET signal [81]. Droplets with high FRET signals were then sorted and collected using sorting chips, while droplets without signals were discarded.

**Figure 5:**
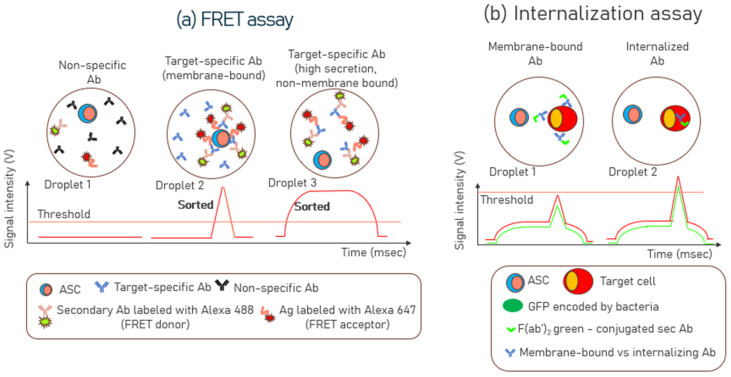
Schematic of (**a**) FRET assay: target-specific antibodies bring the FRET donor and acceptor nearby, giving rise to two different kinds of FRET signals—Droplet 2 (membrane-bound) and Droplet 3 (high secretion, non-membrane specific); (**b**) internalization assay: two scenarios with membrane-bound antibodies in Droplet 1 and internalized antibodies in Droplet 2. Secreted antibodies are labeled with green fluorescent secondary antibodies, and target or reporter cells are labeled with a Deep Red Cell tracker. Compared to antibodies on the surface of the cells, internalized antibodies resulted in a higher green fluorescence peak above the sorting threshold.

A distinct advantage of using the FRET assay is that, as the FRET reagents (donor and acceptor molecules) are uniformly distributed in all droplets, it circumvents the fundamental limits of co-encapsulating beads or reporter cells with the ASCs imposed by Poisson statistics [30]. Another distinctive characteristic of the single-cell FRET assay is the ability to simultaneously differentiate the secreted membrane-bound and non-membrane-bound Ab fractions produced by individual droplet-encapsulated cells [81]. Droplets with a narrow fluorescence peak near the cell surface indicate that most antibodies are membrane-bound (Figure 5a—Droplet 2). Droplets with high and uniform FRET signal throughput in the droplet volume indicate a high level of secreted antibodies (Figure 5a—Droplet 3).

### 3.4. Internalization Assays

Internalization assays are useful antibody discovery tools when developing therapeutic antibodies like antibody-drug conjugates (ADCs). These assays identify antibodies that, upon binding to their target on the cell surface, are effectively internalized into the cell—a crucial characteristic for delivering cytotoxic payloads or modulating receptor functions [83,84]. Internalization assay experiments also provide valuable insights into antibody-dependent cell-mediated cytotoxicity (ADCC) for Human Immunodeficiency Virus (HIV) and Simian Immunodeficiency Virus (SIV) infections. Although ADCC primarily functions as a cell surface mechanism, the internalization of envelope glycoproteins (Env) on the surface of HIV and SIV-infected cells reduces Env surface expression, thereby allowing these virus-infected cells to evade ADCC. Thus, internalization assays are critical for understanding the antibody-dependent cell-mediated cytotoxicity (ADCC) of virus-infected cells [85]. Additionally, these assays are essential for understanding the delivery of antibodies to individual cells via cell-penetrating peptides [86]. Previously, it has been shown that the internalization of antibodies by GFP-expressing bacteria, which are secreted by activated memory B cells, could be visualized within droplets via fluorescence microscopy [87]. In the context of improving the detection sensitivity, pHrodo, a pH-sensitive fluorescent dye, has been used to make engulfed apoptotic cells detectable due to the increased post-phagocytic light emission [88]. pHrodo emits light in the red range with increased intensity as the environmental pH decreases. In a neutral pH environment, pHrodo’s fluorescence is minimal, but it becomes brighter in the acidic environment of phagosomes within phagocytes. This property makes it a valuable tool for studying phagocytosis using fluorescent microscopy or flow cytometry. This is a valuable tool for phagocytosis studies to evaluate the internalization of apoptotic cells. Microfluidic droplets can also differentiate between membrane-bound and internalized antibodies for cell-based assays, depending on the concentration of the localized antibodies on the membrane and within the target cells. This is shown in Figure 5b, which was highlighted in the supplementary note of [87]. In another study [89], microfluidic droplets were also used to investigate the uptake of gold nanoparticles (AuNPs) in single cells. AuNPs can be used as carriers to deliver drugs directly into cells, enhancing the efficacy and targeting of treatments, particularly in cancer therapy.

### 3.5. Neutralization and Infection Assays

**Figure 6:**
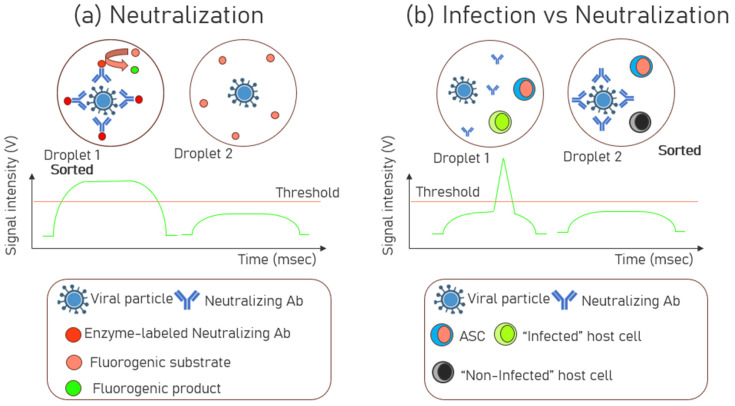
Schematic of (**a**) neutralization assay: Only Droplet 1 has the viral particles bound to the neutralizing antibodies. The unbound antibodies present in Droplet 2 were removed by ultrafiltration. Finally, the enzymes labeled on the neutralizing antibody would convert the fluorogenic substrate into its product in Droplet 1, which would increase the signal intensity above the threshold and would be sorted. (**b**) Infection vs. neutralization assay: two scenarios using droplets encapsulating viral particles, host cells, and ASCs. Unlike Droplet 2, the Infected host cells in Droplet 1 expressed GFP (green fluorescent protein), indicating viral infection. In Droplet 2, neutralizing antibodies bind to the viral particles, mitigating their pathogenic properties. Consequently, the host cells do not become infected or express GFP, and these droplets would be sorted.

Droplet microfluidics has been utilized to screen and sort antibodies capable of neutralizing infections caused by viral particles, such as Human Immunodeficiency Virus (HIV), by targeting specific epitopes [90]. First, the viral particles were incubated with enzyme-labeled antibodies, to allow for binding of antibodies of potentially neutralizing viral epitopes. The Abs were enzyme-labeled for detection purposes, which leveraged the conversion of a fluorogenic substrate into a fluorescent product for fluorescence detection. After incubation, the unbound Abs were removed (washed off) by ultrafiltration to capture only the Ab-bound viruses. Thereafter, all the Ab-bound and unbound viral particles were encapsulated with a fluorogenic substrate. This is shown in Figure 6a where Droplet 1 represents Ab-bound and Droplet 2 represents the unbound-viral particles. Droplets containing virus particles with bound antibodies, became highly fluorescent and were sorted (Droplet 1 in Figure 6a). These bound antibodies are regarded as broadly neutralizing antibodies as they can neutralize the virus by binding to its critical regions, thereby preventing the virus from infecting healthy cells. After sorting, the viruses were recovered from the droplets, and the env genes were amplified by RT-PCR. This process enabled the identification of the genetic sequences of the envelope proteins in the viruses that exhibited the neutralizing epitopes. A single sort resulted in a 1900-fold enrichment of viral particles displaying neutralizing epitopes.

In a separate study [91], Ab-secreting hybridoma cells were first encapsulated in droplets, then merged with other droplets containing viral particles to neutralize the viruses. Finally, these droplets were again merged with host L2 cells, potentially allowing the virus to infect them. The infected host cells expressed GFP (green fluorescent protein), indicating viral infection. The absence of GFP fluorescence suggested that the antibodies had already neutralized the virus. The primary goal of this study was to demonstrate the system’s ability to identify neutralizing antibodies by monitoring fluorescence intensity, which indicated whether the host cells were infected. Another study also adopted a similar working principle [43] in which host cells were used to measure the degree of infection. When the virus infected the host HEK293T cells, the virus replicated within these cells. The fluorescent protein was expressed during replication, causing the infected host cells to emit fluorescence. The intensity of the fluorescence signal was correlated with the degree of infection. Droplets with low fluorescence signals (indicating neutralization and no infection) were collected for further analysis. In Figure 6b, Droplet 1 represents infection, and Droplet 2 represents neutralization.

The tables presented in this review provide a detailed compilation of various assays utilized in the realm of droplet microfluidics, highlighting their critical roles in advancing antibody discovery. Table 1 enumerates the binding assays that have been documented in the literature, showcasing the incorporation of diverse microfluidic platforms such as droplet sorters and barcoded microarrays, along with methodologies like Fluorescence-assisted Droplet Sorting (FADS) and Fluorescence-assisted Cell Sorting (FACS). Table 2 focuses on FRET-based assays, illustrating how different platforms have been leveraged to facilitate these sophisticated molecular interactions. Finally, Table 3 presents an array of functional assays reported in the literature, demonstrating the versatility and adaptability of microfluidic platforms in streamlining antibody screening and functional characterization. Together, these tables underscore the transformative potential of microfluidics in enhancing the speed, precision, and efficiency of antibody discovery.

## 4. New Developments for Improved High Throughput Screening

### 4.1. High Cell and Bead Encapsulation in Droplets

The number of co-encapsulated cells and beads per droplet produced by various droplet generation devices, such as T-junctions, flow-focusing junctions, or co-flowing junctions, can be estimated by Poisson statistics [27,36]. The limitations of Poisson statistics-driven encapsulation in microfluidic droplets include a tendency for a high proportion of droplets to be either empty or contain multiple cells or beads, which can reduce the efficiency and precision of single-cell or single-bead assays. This randomness often necessitates higher input concentrations to achieve a desired level of occupancy, resulting in waste of reagents and increased costs. The Poisson distribution is given by $p\left(k,\lambda \right)=\frac{\lambda^{k}e^{-\lambda }}{k!}$ -> Equation (1), where p(k, λ) is the probability of finding *k* particles (cells or beads) in a droplet, and *λ* is average number of particles (cells or beads) in a droplet volume. As the encapsulation rates of both beads and cells in droplets are mutually independent of each other, the probability of encapsulating both *k*_1_ cells and *k*_2_ beads in a droplet is given by the product of their individual probabilities, i.e., $P_{both}=\frac{\lambda_{1}^{k_{1}}e^{{-\lambda }_{1}}}{k_{1}!}$x$\frac{\lambda_{2}^{k_{2}}e^{{-\lambda }_{2}}}{k_{2}!}$, where *λ*_1_ and *λ*_2_ represent the mean number of cells and beads per droplet, respectively. For instance, in [96], when 50 pL droplets encapsulated with cells and beads were used for bead binder-based antibody discovery, the cells and beads concentrations taken were ~1.4 × 10^6^ (cells/mL) and ~4 × 10^6^ (beads/mL), respectively. As there were two aqueous inlets (one for cells and another for beads in equal proportions) for generating the droplets, the droplet volume per particle translated to (50/2) pL = 25 pL. Based on Equation (1), the average number per droplet is computed to be: *λ*_1_ (mean number of cells/droplet volume) = 0.04 and *λ*_2_ (mean number of beads/droplet volume) = 0.10. Using Equation (1), The percentages of droplets containing single cells and beads are 3.4% and 9%, respectively. Hence, the percentage of droplets containing single cells and beads is their product, which becomes 0.306%.

To overcome the Poisson distribution, Dean force-coupled inertial ordering of single cells in curved, spiral microchannels was introduced using HL60 and K562 cells [97]. This increased the encapsulation efficiency of single cells to 77% by focusing the cells into a single line with equal spacing. Another advantage of inertial ordering shown in this work was that individual cells could be encapsulated at throughputs of magnitude more than without ordering. Since then, spiral microchannels have been used to index both cells and beads separately into droplets [37,98,99]. To help drain excess aqueous phase from the spiral microchannel and reduce the distance between particles, a recent design added a waste outlet with optional serpentine channels to control flow resistance. Other publications overcame the Poisson distribution by performing an active, on-demand encapsulation of fluorescent beads [44] and cells [100] using surface acoustic waves (SAW). An interdigital transducer was activated upon fluorescent signal detection to generate a SAW. This wave displaced the bead or its surrounding fluid [44,100] to generate a droplet encapsulated with the fluorescent entity.

### 4.2. Microfluidics Workstation Design Considerations for High-Throughput Droplet Sorting

**Figure 7:**
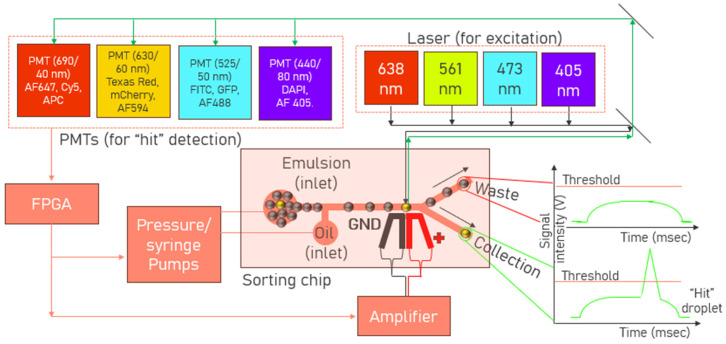
Block diagram of the sorting workstation, comprising four commonly used laser excitation wavelengths (405 nm, 473 nm, 561 nm, 638 nm) and four corresponding PMTs for collecting some of the most popular fluorophores. The sorting chip has been shown with its emulsion and spacer oil inlets, and the waste and collection outlets. Whenever “hit” droplets are detected by any of the PMTs with its signal intensity above a user-defined threshold, a field programmable gate array (FPGA) triggers the sorting pulse via the high voltage amplifier. Droplets, electrodes, channels not drawn to scale.

In 2023, the first detailed step-by-step guide for constructing and setting up a microfluidic workstation for high-throughput droplet analysis and sorting was provided by Jatin et al. [101]. The goal was to democratize the technology and make it more accessible to a broader range of laboratories. It included instructions for assembling the workstation, information on the design and capabilities, troubleshooting tips, and for example workstation applications. The document provided a step-by-step guide for incorporating an integrated circuit known as a field programmable gate array (FPGA) to enhance the computation capacity beyond traditional desktop computers. FPGA modules are needed for fluorescence-activated droplet sorting workstations as the real-time analysis of voltage signals from multiple photomultiplier tubes (PMTs) requires a computational infrastructure that supports high sampling rates (exceeding 50 kHz). FPGA is used for high-speed data acquisition and real-time processing, essential for droplet sorting operations. The document also provided other resources, such as Computer-Aided Design (CAD) files for hardware machining and the microfluidic chips and software (LabVIEW-based) codes for the process control (although the source files have not been provided). Previously, the same group also developed the iSort method which automated high-throughput droplet screening and reduced false positives by using impedance analysis to monitor droplet trajectories in real-time [102]. A block diagram of the sorting workstation is provided in Figure 7.

Despite the workstation comprising multiple components, no publication has yet emphasized the critical significance of selecting appropriate excitation lasers and photomultiplier tubes (PMTs) for detection. A list of different lasers and PMTs used in the literature is shown in Table 4 and Table 5, respectively. These two components represent some of the most critical elements in the overall configuration, as workstations frequently need to accommodate multiple wavelengths for both excitation and detection of different fluorophores. Incorporating the right laser is crucial as it dictates the sensitivity and resolution of the fluorophores used in the biological assay. A good laser must provide a stable power output over extended durations. It should have a narrow linewidth, reducing spectral overlap between fluorophores and improving the signal-to-noise ratio (SNR) readout. The laser beam must be perpendicular to the direction of the fluid flow in the microchannels for effectively exciting different fluorophores within the droplets. Moreover, the laser spot should only engulf one droplet at a time within the microchannels, minimizing the overlap with the neighboring droplets and the background signal from its surroundings. Hence, the laser beam diameter needs to be adjusted according to the size of the droplet, which requires screening. If the beam diameter is too large or too small in comparison to the droplet size, the laser beam’s diameter can be arranged to pass through various optical lenses (cylindrical lens, plano-convex lens, etc.) as shown in Table 4.

### 4.3. Scope of Automation

Automated drug discovery platforms can accelerate compound discovery and optimization in the drug discovery process. Recently, tissue-processing technologies on microfluidic platforms [107,108] have been developed, which reduced the processing times needed to break down different tissues into single-cell suspensions and resulted in up to 10-fold high single-cell recovery. Such platforms are capable of reproducibly isolating splenocytes of harvested spleens from immunized animals and can subsequently be seamlessly integrated with robotic and automated sorting systems, referred to as RAD or “Robotic Automation of Droplet” microfluidics [109]. Such systems eliminate the need to freeze cells for long-term storage, thereby enabling immediate downstream applications that require fresh cells, such as single-cell RNA sequencing [110]. Secondly, the automation of microfluidics can be crucial in accelerating the repetitive aspects of drug discovery by eliminating manual intervention in multistep workflows that require compound synthesis, screening, and optimization in highly controlled experiments [111]. Thirdly, microfluidic devices can also perform high-throughput screening of compounds by parallelizing assays. This allows for testing thousands of compounds in a short period, reducing the time and cost required for screening. It can also integrate different kinds of detection tools that can generate laser/PMT-based fluorescence [101] readouts, electrode-based impedance [112] readouts, infrared [113], mass [114] or absorption [66] spectroscopy, AI-guided and image-based micro-object classification in droplets [115], etc., for real-time monitoring of mechanical, biochemical, and cellular processes [116,117]. The choice of on-chip detection tools may vary depending on the application and the specific research or analysis. These sophisticated detection mechanisms enable continuous monitoring of mechanical, biochemical, and cellular processes, providing rapid feedback and enhancing the optimization of drug candidates. A key benefit of microfluidic technology is its efficient use of minimal samples and reagents, crucial for conserving expensive compounds and limited or precious samples. This reduction in resource consumption decreases experimental costs and allows for more sustainable and versatile screening approaches. Overall, the integration of microsensors and/or automation of microfluidics holds tremendous potential to accelerate drug discovery, offering faster, more cost-effective, and precise experimental workflows.

## 5. Commercial Landscape

On-chip Biotechnologies (Link: https://on-chipbio.com/), incorporated in 2005, was the first company to launch a microfluidic chip-based cell sorter on the market. Initially, the company was set up to perform damage-free gentle on-sorting of delicate cells without using droplets [91]. Still, with the growing popularity of droplets used as microvessels for single cells, it also began offering products that could also perform on-chip droplet generation and sorting. Using picoliter droplets, the first startup specializing in developing single-cell analysis systems and technologies was Sphere Fluidics, founded in 2010 [23] (Link: https://spherefluidics.com/) in Cambridge, United Kingdom. This company’s sole objective was to develop commercial opportunities using picoliter droplets to screen and characterize single cells rapidly. Its sorting benchtop instrument is called Cyto-mine [80], and it is the first fully integrated platform of its kind that can perform several functionalities, such as encapsulation of samples into droplets, incubation of the droplets, and sorting and dispensing of the “hit” droplets to microwell plates all within one instrument. Another startup that uses pico-droplet technologies specifically for making single-cell resolution-based immunotherapies is HiFiBio (Link: https://hifibio.com/), founded in 2013. Their core technologies are Drug Intelligence Science (DIS) [118], which combines single-cell technology with AI/ML to enhance the probability of success for drug discovery and development, and Celligo, a droplet microfluidics-based HT sorter for screening IgG-secreting cells [87]. A noteworthy aspect of Celligo technology is its ability to maintain the crucial linkage of VH and VL (heavy-chain and light-chain variable region) pairs [87]. This was achieved by re-compartmentalizing sorted cells with barcoded hydrogel beads for reverse transcription and sequencing. This innovation was applied to the exploration of IgG repertoires from mice immunized with various antigens, including the vaccine target Tetanus Toxoid (TT), the multifunctional enzyme Neuroleukin/Glucose-6-Phosphate Isomerase (GPI), and the membrane-bound cancer target Tetraspanin-8 (TSPAN8). Using Celligo, at least 20% of the VH-VL pairs were identified from the recovered IgG-secreting cells (plasmablasts and plasma cells).

Yet another prominent startup is Velabs Therapeutics, founded in 2017 at the European Molecular Biology Laboratory (EMBL) in Germany. The company, which later became Veraxa Biotech (Link: https://www.veraxa.com/), was initially set up to specialize in microfluidics-based technologies for rapidly screening functional antibodies. It has now broadened its focus beyond functional antibody discovery to other therapeutic modalities like Antibody-Drug Conjugates (ADCs) and conditionally activated bispecific antibodies [119]. ADCs [120] have become increasingly popular in recent years and are constructed with three main elements: a monoclonal antibody designed to target a particular cancer-associated antigen with high affinity and specificity; an anti-cancer medicine as a payload, like a chemotherapy drug, and a “linker” that cleaves off that payload inside cancer cells. The main challenge [121] associated with ADC development is that it requires the ADCs to have both high affinity and specificity towards a target antigen, thereby eliminating the payload cytotoxicity to healthy tissues. Another challenge is that high internalization is required to be functional. Once an antigen is identified, the first step in an ADC development project is to conduct extensive screening to acquire a few suitable monoclonal antibodies for specific target recognition. As Veraxa is equipped with an HT droplet-based microfluidic system, it is well-placed to conduct such extensive screening for ADC development. Another notable mention in this field is Atrandi Biosciences (Link: https://atrandi.com/). They focus on droplet microfluidic tools to enhance our understanding of cellular behaviors, interactions, and functions. Two more early-stage startups founded recently for antibody discovery using HT droplet microfluidics are Shennon (Link: https://www.shennonbio.com/) and Aureka Biotechnologies (Link: https://www.aurekabio.com/), which functionally identify and sort out antigen-specific T/B cells from the 100 billion T/B cells in the body.

## 6. Current Challenges

### 6.1. Translation from Benchtop to Droplet Format

Transferring a bioassay into the droplets of a microfluidic device is not a straightforward task. This is because macro-scale experiments cannot directly be translated from benchtop to the droplet format due to differences in reagent volumes (milliliters to microliters), single-cellular resolution, and increased surface area to volume ratios. For instance, in [74], a bead binder assay was reported, which used a concentration of 6.5 µg/200 µL of fluorophore-conjugated secondary/detection Abs (115-485-006, Jackson ImmunoResearch, Cambridgeshire, UK). This computes to a concentration of 32.5 µg/mL. Although this item (115-485-006, Jackson ImmunoResearch) is no longer commercially available, similar replacement items such as (115-546-006, Jackson ImmunoResearch) are still available. The recommended working concentration or dilution range for (115-546-006, Jackson ImmunoResearch) is 1:100–1:800 from a stock concentration of 1.5 mg/mL, which computes to a range of 15–1.875 µg/mL. This is much lower than the original concentration of 32.5 µg/mL used in [74]. This implies that researchers need to optimize significantly to implement the right concentration of detection Abs from a bulk assay to microfluidic droplets. In the context of a bead binder assay, if the concentration of fluorophore-conjugated secondary/detection antibodies is too high, the background signal of the bioassay will be too strong. And as a consequence, it will not be possible to distinguish between empty droplets and those containing beads and/or cells. Similarly, if the concentration is too low, Ag-coated beads within the “hit” droplets will not induce a significantly strong SNR due to the insufficient localization of fluorophore-conjugated detection antibodies onto its surface. The concentration of the fluorophore-conjugated detection antibodies in the droplets must be adjusted to be lower than the total binding capacities of the beads encapsulated in the droplets.

### 6.2. Defining and Identifying “Hit” Droplets

In high-throughput screening, a “hit” droplet is defined as one that displays a desired effect or potential activity, encapsulated within a droplet. This is indicated by a signal output that exceeds the background noise readout, as quantified by the SNR. A higher SNR above a user-defined threshold can indicate a strong binding Ab-Ag affinity (in case of a binding assay), functional response from reporter cells due to Ab-secretion from ASCs (in case of a functional assay), donor–acceptor proximity (in case of a FRET assay), etc. To identify the “hit” droplet from the remaining “non-hit” droplets, the SNR of the fluorescence signal from the “hit” droplets must be sufficiently distinguishable in the form of detectable PMT signals. In the case of fluorescence-based SNR readouts, the “hit” rate should also be validated from fluorescence microscopy images acquired via control experiments. It is imperative to set up control experiments to establish a baseline before screening valuable biological samples. Moreover, the system’s optical detection sensitivity must be established to compare minimum SNR levels between “hit” and surrounding droplets, which is needed to generate detectable fluorescent signals. Proper alignment of the laser beam onto the droplets and fluorescence emission signal from the droplets onto the PMTs is essential to enhance the optical sensitivity [101]. The absence of proper optical alignment can result in weakened excitation of the fluorophores within the droplets, leading to decreased fluorescence signal intensity. This reduction in signal can impair the detection and differentiation of “hit” droplets from non-hit ones. Hence, maintaining proper optical alignment is crucial for optimizing the performance and accuracy of droplet sorting workstations. Finally, to eliminate batch-to-batch variation and produce reproducible data, the incubation periods of the droplets should also be established to ensure all reaction mechanisms within droplets can produce sufficiently strong detectable read-outs before analyzing the signals.

### 6.3. Addressing Inter-Droplet Diffusion Reduction

Reducing the inter-droplet diffusion and leakage of small molecules from the droplets is crucial for ensuring the reliability and accuracy of fluorescence-activated droplet sorting (FADS) systems. If small molecules, including fluorescent markers or assay components, leak out of droplets, the concentration of these molecules within the droplets will decrease over time. This can alter the assay conditions and lead to inaccurate measurements or false readings, compromising the reliability of the assay. It can also reduce the SNR, making it more difficult to accurately detect and quantify the fluorescence signals associated with “hit” droplets. Small-molecule release from water-in-oil emulsion droplets is caused by its diffusion into the oil phase via the formation of reverse micelles [122,123]. The concentration of the surfactants in the oil phase is an important parameter related to the formation of reverse micelles. Below the surfactant’s Critical Micellar Concentration (CMC), the surfactant molecules are not present in sufficient numbers to form reverse micelles. These reverse micelles can act as reservoirs for small molecules, allowing them to diffuse from the water phase into the reverse micelle core and then into the surrounding oil phase. This diffusion process releases small molecules from the emulsion droplets, causing leakage and potential cross-talk between droplets. The addition of bovine serum albumin (BSA) [122,124] to the aqueous phase of the droplets has been reported to reduce small molecule transfer by blocking the interface between the oil and the aqueous phase or by changing the partition coefficient by small molecule binding.

## 7. Future Outlook

Drug discovery will significantly benefit from an ultrafast screening technology that can analyze vast libraries of potential antibodies through an iterative and data-driven process. Although microfluidics-derived antibody discovery remains largely in the laboratory and early discovery phases, their translation into preclinical or clinical pipelines is beginning to take shape [125]. The synergy between high-throughput screening (HTS) and generative artificial intelligence (AI), is particularly vital in this context. This dynamic interaction facilitates the efficient identification and validation of novel targets by rapidly generating and refining potential antibody candidates based on large datasets, such as genomic or proteomic databases [126,127]. However, while the integration of AI and HTS technologies holds great promise for antibody discovery, several critical gaps remain. Limitations in current AI models—such as the need for high-quality training data, challenges in interpretability, and uncertain generalizability to novel antigens—must be addressed. Additionally, the sheer scale of AI-generated antibody libraries presents technical challenges for current high-throughput screening (HTS) platforms, which may struggle to validate all in silico candidates efficiently through experimental validation. Integrating and standardizing data between computational and experimental workflows remains a significant challenge, as does the regulatory landscape for AI-designed therapeutics. Addressing these issues will require interdisciplinary collaboration and continued innovation in both computational and experimental methods.

To screen a vast library of potential in silico “hit” candidates, an ultrahigh throughput screening technology like fluorescence-activated droplet sorting (FADS) will be important for accelerating various steps such as: (i) generating ground truths and testing large datasets of antibody sequences, structures, and properties to train computational models and algorithms; (ii) making predictions and finding “hit” antibody candidates by improving their antigen targeting (binding affinity, epitope mapping, and target specificity) and functional properties (immunogenicity, solubility, and pharmacokinetics). Once potential candidates are identified through AI-based generative approaches and HTS (e.g., via FADS and FACS) traditional methods [10,21,22,23] become essential for further refinement. Traditional techniques offer an established framework for validation, ensuring that antibody candidates meet clinical and regulatory standards before advancing to later stages of therapeutic development. They enable detailed characterization and optimization of antibodies, improving binding affinity, specificity, stability, and other functional properties. Overall, while modern technologies expedite initial discovery, traditional methods provide the established roadmap to develop and deploy therapeutic antibodies successfully.

As the challenges of traditional sorting methods like FACS become more evident in high-throughput applications, new technologies such as FADS offer valuable opportunities for improvement. While FACS can sort at speeds of tens of kiloHertz, sorting cells at high rates or pressures in a flow cytometer can lead to cell death due to shear stress as the cells are forced through narrow nozzles [68,128]. There are several advantages of using FADS over FACS. For instance, (i) cells are more protected from colliding with each other and narrow nozzle constrictions in microfluidic droplets compared to bulk processing. Microfluidic droplets provide a physical barrier, shielding cells from external stresses like shear forces, turbulence, and contamination. (ii) In bulk processing, the secreted molecules are greatly diluted. However, microfluidic droplets restrict the diffusion of antibodies by encapsulating immune cells in small droplets and enhancing the SNR due to the containment of secreted molecules in these droplets. In other words, the droplets act as confined compartments, preventing the diffusion of secreted cytokines to neighboring cells. (iii) This confinement also reduces the cross-contamination between secreting and non-secreting cells, which can lead to false-positive and false-negative results in cytokine capture assays [77] performed in bulk. (iv) While FACS has been used with double emulsions [129,130], it may not be appropriate for all applications since double emulsions are generally less stable than single emulsions and, in addition, tend to be more permeable to small molecules, which can leach out of the droplets over time [68]. Moreover, no reported publications have yet shown that on-chip operations, such as pico-injection, electrocoalescence, droplet merging, etc., can be performed using double emulsions. Hence, the scope of automation is limited with double emulsions.

While current commercial platforms have demonstrated impressive throughput and flexibility, persistent challenges remain regarding droplet integrity over extended assay times, device reliability during continuous operation, and reagent compatibility for long-term or multiplexed workflows. Ensuring that droplets maintain their compartmentalization and signal fidelity is essential for reproducible high-throughput screening, especially as workflows scale to millions of assays per run. Additionally, robust automation, seamless integration with upstream sample preparation and downstream data analysis, and the ability to maintain consistent performance across large batches or prolonged campaigns are necessary for industrial adoption. These aspects are not always fully addressed in commercial overviews, which often focus on present capabilities rather than unresolved technical hurdles. Addressing these issues will require continued innovation in microfluidic engineering to realize the full potential of FADS as a cornerstone technology for next-generation antibody discovery and development.

As the complete design and construction details of microfluidics workstations for setting up FADS systems have been provided [101], the number of FADS users is expected to increase globally in the antibody discovery industry and educational institutes. This increased user base underscores the urgent need to overcome the challenges previously mentioned. High-throughput microfluidics allows for manipulating and analyzing thousands to millions of individual cells or molecules in parallel, facilitating comprehensive exploration of vast libraries of potential targets. This capability can significantly expand the “target space” by enabling researchers to investigate a wider array of biological targets, such as proteins, genes, or chemical compounds, more efficiently and in greater detail than traditional methods. FADS, capable of screening and sorting millions of single-cell-encapsulating droplets per hour, enables the rapid screening of large antibody libraries, significantly accelerating the discovery process. The FADS community continues to experience steady growth. As AI-based approaches continue to advance, their synergy with high-throughput screening (HTS), including fluorescence-activated droplet sorting (FADS), alongside traditional validation frameworks, is poised to revolutionize antibody discovery and therapeutic development, paving the way for more efficient and precise medical interventions in the future.

## Figures and Tables

**Table 1:** List of binding assays reported in the literature that incorporated various microfluidic platforms: droplet sorter, barcoded microarrays, etc. [Column 7]. FADS: Fluorescence-assisted Droplet Sorter, FACS: Fluorescence-assisted Cell Sorter.

Immunoassay Strategy [Bead (Material)—Diameter (μm)/Coating]	Cell Types/Populations	Primary Ab/Cytokine	Ag/Capture Ab—Reagent	Detection Ab	Microfluidic Platform	References
Streptavidin-coated polystyrene (PS) beads—diameter 6 μm	9E10 cells secreting IgG antibodies against human c-MYC protein	IgG	Biotinylated Goat anti-mouse IgG	DyLight 488 Affini- Goat anti-Mouse IgG, F(ab’)2 Fragment Specific	Droplet Generation + FADS	Singleplexing [74]
Avidin Coated Particles, diameter 0.9 μm	CD4 + CD25 + regulatory T cells	human IL-10	Biotinylated IL-10 mAb (Invitrogen AHC7109)	rat anti-human IL-10 FITC conjugated antibodies	Droplet Generation + FACS	Singleplexing [92]
Barcode array on glass substrate (width—10 µm, pitch—25 µm)	U937-derived macrophage cells, human tumor cell lines (U87 and SCC6 cells)	(i) TNF-alpha, (ii) MIP-1b, (iii) MCP-1, (iv) IL-8, (v) IL-10	(i) Mouse IgG1, κ/Mab11, (ii) Mouse IgG1 kappa/A174E18A7, (iii) Mouse IgG1, κ/5D3-F7, (iv) Mouse IgG1, κ/H8A5, (v) Rat IgG2a, κ/JES3-12G8	(i) Mouse IgG1, κ/MAb1, (ii) Mouse IgG2B/24006, (iii) Armenian Hamster IgG/2H5 (iv) Mouse IgG1, κ/E8N1, (v) Rat IgG1, κ/JES3-9D7	Droplet-based barcoded microarrays	Multiplexing [93]
PS/biotinylated mAbs specific for IL-2, TNF-a, or IFN-gamma	T cells (4 M/mL) stimulated by phorbol 12-myristate 13-acetate (PMA, 1 µg/mL) and ionomycin (0.2 µg/mL)	IL-2, IFN-gamma, TNF-alpha	anti-CD3, anti-CD69, anti-IL-2, anti-TNF-a and anti-IFN-g-	anti-CD3-PE/FITC, anti-CD69-PerCP^®^(both BD Pharmingen), anti-IL-2-Alexa Fluor^®^ 488, anti-TNF-a-PE and anti-IFN-g-Alexa^®^Fluor 647^®^	Droplet Generation + FACS	Multiplexing [75]
Protein A-coated PS, capture Ab, diameter 5.5 μm	D1.3 Hybridoma cells	D1.3, HyHEL-5, and LGB-1	Rabbit or goat anti-mouse pAb	HEL-Dylight488, HEL-Dylight633 for D1.3; HEL-Dylight488 for HyHEL-5, EGFP for LGB-1	Microfluidic flow channels for trapping beads	Multiplexing [94]
NA	T cells stimulated by phorbol 12-myristate 13-acetate (PMA) and ionomycin	human IL-10	Miltenyi IL-2 capture reagent	Miltenyi phycoerythrin—conjugated anti IL-2 Ab	Parallelized Droplet Generation + FACS	On-cell membrane [95]
Target cells: K562 cells	OKT9 hybridoma cells	IgG	NA	goat anti-mouse IgG Alexa 488 antibody	Droplet Generation + FADS	Cellular Binding (Cell surface) [28]
Target cells: EGFR-positive A431 cells	mAb108 hybridoma cells	EFGR-specific IgG	NA	Alexa 488 AffiniPure Fab goat anti-mouse IgG (H + L)	Cytomine	Cellular Binding (Cell surface) [82]

**Table 2:** List of FRET-based assays reported in the literature that incorporated various microfluidic platforms: droplet sorter, barcoded microarrays, etc. [Column 8]. FADS: Fluorescence-assisted Droplet Sorter, FACS: Fluorescence-assisted Cell Sorter.

Animal Model	Immunogen	Cell Types/Populations	Primary Ab	Donor	Acceptor	Microfluidic Platform	References
Mouse	human TNF-alpha	DG44 CHO cell line	human IgG4	Green fluorophore-conjugated human TNF-alpha	Red fluorophore-conjugated anti-mouse IgG-Fc Ab	Cytomine	[80]
NA	c-myc peptide	9E10 cells secret IgG antibodies against human c-MYC protein	IgG	Alexa Fluor 488-conjugated anti-mouse F(ab’)2-specific pAb	Alexa Fluor 647-conjugated c-myc peptide	Droplet Generation + FADS	[81]
Human, mouse	Tetanus Toxoid	Plasma cells (bone marrow, lymph nodes, splenocytes)	IgG	Goat Anti-Human IgG Fc-DyLight^®^ 488	Goat F(ab’)2 Anti-Human IgG—(Fab’)2 (DyLight^®^ 594), pre-adsorbed	Cytomine	[82]

**Table 3:** List of functional assays reported in the literature that incorporated various microfluidic platforms: droplet sorter, barcoded microarrays, etc. [Column 8]. FADS: Fluorescence-assisted Droplet Sorter, FACS: Fluorescence-assisted Cell Sorter.

Target Cells	Cell Types/Populations	Reporter Cells	Primary Ab	Detection Criteria	Microfluidic Platform	References
K562 cells	NK-92 MI cells (coated with IFN-γ capture reagent)	NA	Anti IFN-γ Ab	APC signal from IFN-γ detection Ab	Droplet Generation + FACS	[77]
NA	OKT3 hybridoma cells	Jurkat-GFP cells	anti-CD3 Ab	GFP signal from reporter cells	Cytomine	[82]
NA	Hybridoma cells expressing Ab, 4E3 which inhibits ACE-1	NA	Antibodies that target and inhibit ACE-1	Droplets with low fluorescence intensity were sorted which indicated the presence of ACE-1 inhibitory Ab	Integrated chip (Generator + fusion + on-chip incubation + FADS)	[76]
NA	K562-Her2 cells (positive control), HEK293FT cells infected with lentivirus	Jurkat/NF-κB-GFP, Jurkat/pIL2-eGFP	Anti-Her2 × anti-CD3 bispecific Ab	Reporter cells’s Cell Trace Yellow signal and K562-Her2 cells’s Cell Trace Violet signal	Droplet Generation + FADS	[78]

**Table 4:** List of different lasers used in microfluidic setups for droplet sorting. OL = Objective lens, CL = Cylindrical lens, PCL = Plano-convex lens.

Manufacturer	Wavelengths (nm)	Power (mW)	Beam Diameter (mm)	Directly Passes Through OL (Y/N)	OL’s Magnification	Droplet Volume (pL)	References
Changchun Dragon Lasers	405	50	1.2	Y	10×, 20×	140	[101]
	473	100	2	Y	10×, 20×		
	561	50	NA	Y	10×, 20×		
Melles-Griot	488	50	0.7	CL -> OL	20×	50	[74]
Omicron (PhoxX+ 488-100)	488	100	0.7–1	CL -> OL	20×	40	[81]
Changchun New Industries (CNI)	473	NA	<1.2	NA	NA	8	[68]
Changchun New Industries (CNI)	473, 532, 640 (aligned)	100	<1.2	NA	NA	270	[103]
Picarro Cyan	488	20	NA	Y	40×	~24	[104]
Newport-Spectraphysics	488	20	1.3 ± 0.3	CL -> PCL -> OL	40×	12	[105]
Omicron (combiner) Laserage GmbH	365 + 488 + 561	NA	0.7	OL	NA	~17	[106]

**Table 5:** List of different photomultiplier tubes (PMTs) used in microfluidic setups for droplet sorting. BW = Bandwidth.

PMT—Model, Company	Sorting Speed (Hz)	Frequency BW (kHz)	Gain	Wavelength Detected (min, max)	Wavelength Detected (peak)	References
H10722-20, Hamamatsu	100–200	20	2 × 10^6^	230, 920	630	[101]
	500					[81]
	1000					[106]
H5784-20, Hamamatsu	200		5 × 10^5^	230, 920	630	[74]
	300					[105]
PMM02, Thorlabs	30,000		5.1 × 10^5^	300, 800	420	[68]
